# Emotional Self-Regulation of Individuals with Autism Spectrum Disorders: Smartwatches for Monitoring and Interaction

**DOI:** 10.3390/s17061359

**Published:** 2017-06-11

**Authors:** Juan C. Torrado, Javier Gomez, Germán Montoro

**Affiliations:** Department of Computer Engineering, Universidad Autónoma de Madrid, Madrid 28049, Spain; jg.escribano@uam.es (J.G.); german.montoro@uam.es (G.M.)

**Keywords:** wearable computing, assistive technologies, affective computing, behavioral monitoring, cognitive disabilities, mobile assistance, ubiquitous computing

## Abstract

In this paper, we analyze the needs of individuals with Autism Spectrum Disorders (ASD) to have a pervasive, feasible and non-stigmatizing form of assistance in their emotional self-regulation, in order to ease certain behavioral issues that undermine their mental health throughout their life. We argue the potential of recent widespread wearables, and more specifically smartwatches, to achieve this goal. Then, a smartwatch system that implements a wide range of self-regulation strategies and infers outburst patterns from physiological signals and movement is presented, along with an authoring tool for smartphones that is to be used by caregivers or family members to create and edit these strategies, in an adaptive way. We conducted an intensive experiment with two individuals with ASD who showed varied, representative behavioral responses to their emotional dysregulation. Both users were able to employ effective, customized emotional self-regulation strategies by means of the system, recovering from the majority of mild stress episodes and temper tantrums experienced in the nine days of experiment in their classroom.

## 1. Introduction

### 1.1. Autism Spectrum Disorders and Emotions

The term “autism spectrum disorders” (ASD) derives from the identification of the formerly called “autism” as a wide group of heterogeneous neurodevelopmental disorders [1]. The most recent diagnostic classification from the DSM-V [2] highlights two main aspects to determine the presence of an autism spectrum disorder in an individual. On the one hand, pervasive deficits on communication and social interaction must be found; on the other hand, there would be presence of repetitive patterns of behavior, activities and interests, which manifest through several symptoms. This heterogeneous nature of the autism makes the diagnosis assessment complex and expensive, and it is made later in life although the symptoms are shown in the early childhood, based on descriptions and observations of behavior [3,4]. Although these symptoms are varied and multi-faceted (stereotypy, inflexibility, rituals, restricted interests, obsessive behaviors, sensory disturbances…), experts summarize them into “lack of ability to control actions”, also called executive dysfunction [5,6,7].

#### 1.1.1. Self-Determination and Executive Dysfunction

Executive dysfunction comprehends effects on planning and organization skills, impulse control, inhibition of inappropriate responses and flexibility of thought and action [5]. Concretely, executive dysfunction leads to practical difficulties in the organization and sequencing of steps within a task (even identifying the starting and ending points of the task), and difficulties in behavioral and emotional regulation. This lack of emotional regulation of children with autism spectrum disorders is represented by many, significant problems in the area of emotional lability: frequent temper outbursts, tendency to cry, rapid mood changes and a tendency to be frustrated if demands are not easily met. The alexithymia associated with these problems also relates to other problems at externalizing behaviors, having temper outbursts with them and explosive behaviors [8]. The common route for emotional regulation followed instinctively for people without autism spectrum disorders is defined as “some automatic or intentional modifications of the individual’s emotional state that promotes adaptive or goal directed behavior” [9]. Outbursts, aggressions and meltdowns are the consequence of people with autism spectrum disorders’ failure to employ adaptive emotion regulation strategies. Mazefsky also remarked the long-term effects of the lack of emotion regulation of people with autism spectrum disorders, which include irritability, anxiety, depression and impulsivity.

#### 1.1.2. Do I Need Assistance?

Alexithymia is defined as the disability to identify and describe feelings [10]. It also involves difficulties on distinguishing feelings from body sensations of emotional arousal and impaired symbolization. If the user is intended to self-regulate emotionally, these emotions should be recognized and managed, so individuals with ASD have to receive assistance in this matter. Many aspects of their daily life become triggering stimuli to behavioral disturbances. For example, Ashburner [8,11] speaks about the stress sources of individuals with Asperger’s syndrome and high functioning autism that are studying in a mainstream school: they often get bullied, or frustrated by academic underachievement, and in many cases, the response of the school staff to their child’s challenging behaviors is also deficient. This combination of alexithymia and high sensitivity evidences the need of intervention in the emotional sphere of people with autism spectrum disorders.

### 1.2. Autism Spectrum Disorders and Technology

#### 1.2.1. Acceptance and Social Stigma

The use of technology to aid people with cognitive disabilities is widespread and rich in the literature, though there is also a factor of success that is not very regarded in the field, which is called “acceptance” by several authors [12,13,14,15]. Acceptance has to do with the relation between the user and a technology product: although the product is very reliable, robust and their effects proven, actual success depends on the usage once it is deployed within the user’s daily life. Acceptance derives from the following factors: implication of the family in the learning process, their previous level of technology usage, the very user’s level of technology usage, the usability of the product, actual satisfaction of a certain need, intrusiveness, reliability, ease of configuration, maintenance, replacement and update [14], and a very significant one, the reduction of social stigma [16,17]. This term refers to the difference implied in a user having certain product and others not having it. Even if an individual does use the product and others in his environment do not, the social stigma can be reduced, depending on the shape, usage and level of presence of the product. For instance, if an individual wore a chest band that monitored some signals, and it had to be worn over the coat, it would be clearly visible, so the individuals would notice a clear difference between them and the others. However, if the same individuals could wear the same device but inside the coat, or a smaller band, the social stigma would be reduced although it is a product used just by them. Also, it is quite clear that social stigma is reduced to its minimum when the assistive technology is deployed in a device used by the mainstream, like smartphones, tablets or computers [18]. In the literature, acceptance is treated as its opposite factor, which is the abandon rate [19,20,21]. Abandon causes are usually framed into the usability area, so many researchers attribute them to the low suitability of standard usability decisions to these users.

#### 1.2.2. The Role of Caregivers and Family

The lack of emotion regulation in people with autism spectrum disorders, as with cognitive impairment in general, causes some behaviors that may be challenging for their family and people in their environment (aggression, self-injury, outbursts, defiant attitude…). Stress coping of familiars of individuals with autism spectrum disorders is well-documented in the literature [22,23,24], and the increasing number of individuals with ASD in the recent decades also remarks the need of support for the families [3]. The challenge of individuals with autism spectrum disorders’ behavior is also demanding for their caregivers, even if they are specialized on the matter. When it comes to perform intervention strategies with technology, the above-mentioned tasks of updating, maintenance, installation and replacement are responsibility of family and caregivers, which also increases their stress levels. In conclusion, the role of caregivers and family can be eased by making the intervention for emotion regulation more effective (so the associated behavior becomes easier to cope with) and reducing the abandon rate by easing the technology management tasks. For instance, a considerable approach would be using popular, well-known devices for intervention, which does not need to learn new technology management. Social stigma reduction, familiarity, simplicity of usage and future prospects (as will be explained later) will be the criteria for technology selection in this paper.

### 1.3. Autism Spectrum Disorders and Intervention Strategies

The reason behind intervention in people with autism spectrum disorders is to increase the independence of the individual, as well as to reduce the features of autism to its minimum [25]. Proper interventions facilitate development and learning, promote socialization, reduce maladaptive behaviors and support families, but they tend to be multi-faceted [26] and costly, because the pervasive nature of the disorder, which requires interventions across lifespan [3]. There is a big, growing corpus of intervention strategies reviewed in the literature, and well documented evidence-based practices [26] designed in order to perform improvements in behavior, like discrete trial training (DTT) [27], parent-implemented interventions [28], peer-mediated instruction intervention (PMII) [29], picture exchange communication systems (PECS) [30], pivotal response training (PRT) [31], social narratives [32], video modeling [33], self-management [34] and positive behavior support [35]. This last one also includes the following techniques: functional behavior assessment (FBA) [36], stimulus control [37], response interruption [38], functional communication training [39], stimulus extinction [40] and differential reinforcement [41].

#### Procedures and Media

Given the whole list of autism spectrum disorder intervention strategies for improving behavior, some pertinent questions arise. How are they implemented? What are the main constraints of each one to be implemented? What materials do they require, and are they always available and feasible to acquire? These questions have to do with caregivers, materials and technology. Unavoidably, mentioned strategies like PII or some implementations of PRT that require in-situ role playing, involve people around the individual, and the application of these intervention strategies are constrained by their availability. This availability involves an economical and logistic effort for the individual’s family and environment. However, this effort can be lessened in other strategies that classically uses the support of the people around the individual with autism spectrum disorder, but in which technology usage would be significant and affordable. What does it mean to be “significant” to introduce technology in an intervention strategy, or to adapt its materials to include technology? Some of the stated interventions (in fact, the most recent ones) are intrinsically technological, like video modeling; but the materials involved in some of the others (pictograms, prompted instructions, reminders, exercises) can be implemented with technology in order to ease costs and logistic needs, and adding features only available with technology like pervasiveness, increased outreach, performance and efficacy. Either the technology is introduced in an intervention strategy or the intervention is intended to use technology when designed, an unavoidable process should be the deep study of the interaction between the user and the technology. This relates to the previous matter of assistive technologies, and given the strict features of individuals with autism spectrum disorder, the interaction paradigm must be designed taking into account new ways of interaction, and considering possibilities of new devices that also guarantee acceptance, ease the availability of the assistance and reduce costs.

### 1.4. From Inward State to Interaction

The literal meaning of “human computer interaction” relates to all action performed between an individual and a machine, explicit or implicit. However, the implicit channel of human computer interaction has been poorly explored, despite of having some early literature predicting that “(…) the availability of processing power and advanced sensing can enable a shift in HCI from explicit interaction (…) towards a more implicit interaction based on situational context.” [42]. This shift seems yet to be performed, because HCI nowadays keeps that deeper concern with the explicit channel at the expense of the implicit one. However, several growing research fields have this idea at their basis: context-awareness [43], ambient intelligence [44] and wearable technologies [45], which are strongly interconnected. Assistive technologies for cognition [46], of course, have been nurtured from these research fields. The main reason is that implicit interaction works in some ways as a substitute of the cognitive impairment of the users by which this technology is intended to be used. In fact, the alexithymia associated to users with autism spectrum disorders requires a technological intervention that eases this deficit in the same way. Implicit interaction associated with the reading of the inward state of the user is strictly related to another prolific research field: affective computing [47]. The parallel growing of wearable technologies, whose innovations crystallized into commercial, widespread devices used as well by the mainstream and the researchers, helped the affective computing area to grow spectacularly in recent years [48]. Here we will discuss the elements from these fields that concern autism spectrum disorder features and the assistive technologies involved.

#### 1.4.1. What Is Inward State?

It is said that the main point of wearable devices is their capacity to sense simultaneously the outward and the inward state of the users, that is to say, their specific condition. The outward refers to the external signals that come from anything that does not refers to the user (temperature, humidity, sounds, movements, radio signals, etc.) which is the classical sensing approach. However, the opposite one has been the mainstream in fields of medicine-related technologies [49,50] where the user state is the object of study, ergo sensing. Thus, bringing it to HCI issues can be conceived as an import, where technology had to be redefined, and paradigms of interaction created [51]. Recently, wearable technology, as the main expression of inward state processing in HCI, has been giving feedback to health informatics [52], adapting the paradigms to new therapies and studying the use of innovative devices in the treatments and everyday lives of patients [53,54]. The inward state does not only relate to physiological data (although is the main data income), but also to other measurements that provide information of the user: activity recognition [55] by user’s movements, steps, postures, location [56], face expression [57], gestures [58], tempo-spacial relationship between them and recorded habits, through user reporting [59,60] or pattern recognition of the aforementioned measures [61]. It is necessary to understand that, as with the outward, inward state does not refer to the very measures or signals, which are mere, concrete descriptors, but to the abstract inner situation of the user in a certain moment, which is to be approached and parametrized through these signals. For instance, in an intuitive way, we could say that the inward state referred to as “stress” may be associated with high levels of heart rate variability, elevated respiration rate, certain face expressions and increased movement of upper and lower limbs; that is to say, the abstract, inward state of “stress” can be approached as a set of measurable parameters in a given moment using technology.

#### 1.4.2. Wearables as Translators of Inward State

This parameterization of the inward state is what relates to the “implicit interaction” that we described in the earlier sections: users are not pressing a button, navigating through an interface nor thinking about the technology involved when they are changing their inner state. However, the physiological, ergonomic and circumstantial effects of this change may become the input of a wearable system that processes this information and gives a certain output [62]. This may be aimed to help the user managing this affect change, or create a log file of inner states, so the user can receive some kind of feedback later, for example. Not all wearable-based systems rely on this inward modeling paradigm: not even all wearable-based systems for individuals with cognitive impairment-related problems; an example of this is the wrist-worn prompter by Kearns et al. [63], which is a reminder and scheduler of daily-life tasks, and only needs the time, date, and some explicit interaction from the user (it is even programmable by other individuals remotely): the whole input is external to the user. SPARK, the smartwatch system by Sharma [64], it is also a reminder for individuals with Parkinson disease that helps them remembering their medication (again, no inward use). Inward state is used by Wile [65] and Shin [66] for monitoring purposes on individuals with Parkinson disease and dementia, respectively. Inward state has been also explored in terms of habit detection [67] and mood recognition during activities [68]. In fact, wearables are more inward-oriented devices. The intention of making them wearable is for them to be able to sense signals and events happening on the body of the user. Though the explicit channel of interaction is being explored in the literature [69], the implicit interaction of user and wearable is yet to be more broadly explored, its advantages outlined through newer literature corpus in the fields of affective computing [70] and activity recognition [71]. 

#### 1.4.3. Smartwatches: Towards Acceptance

This “implicit interaction”, described above, seems adequate to supply the executive dysfunction of people with autism spectrum disorders, regarding their behavior problems. However, as we stated previously, the wearable device that assists the users in their emotional self-regulation process has to achieve a high level of acceptance, because it is supposed to be worn pervasively, and the assistance received in any moment of the day. Most of the devices proposed by the literature are specifically designed for particular cases: Witt proposes a smart glove to study implicit interaction [62], Shuzo uses an ear-worn system to study eating habits [61], Lee describes an intelligent belt for activity recognition [56] and Kearns proposes a non-commercial smartwatch, integrated into a smart environment [63]. These artifacts are problematic in terms of acceptance because of the differentiation they suppose on the individual when they are worn. The problem of differentiation goes beyond intrusiveness although, in some cases, goes together: are Kearns’ smart gloves supposed to be worn in warm days? Shuzo’s ear-worn allows the individual to use headphones at the same time for listening music? For example, Lee’s belt cannot be considered a highly intrusive wearable: belts are light, clothing components, fixed to the waist, and this wearable system just adds some electronic components to the belt, although their placement and size, regarding production levels, is arguable. However, other people establish a difference by plain sight between an individual with the smart belt and another without it (or a standard belt). In terms of acceptance, detecting this difference easily is very negative, and might become a cause of technology abandonment [14]. 

**Figure 1:**
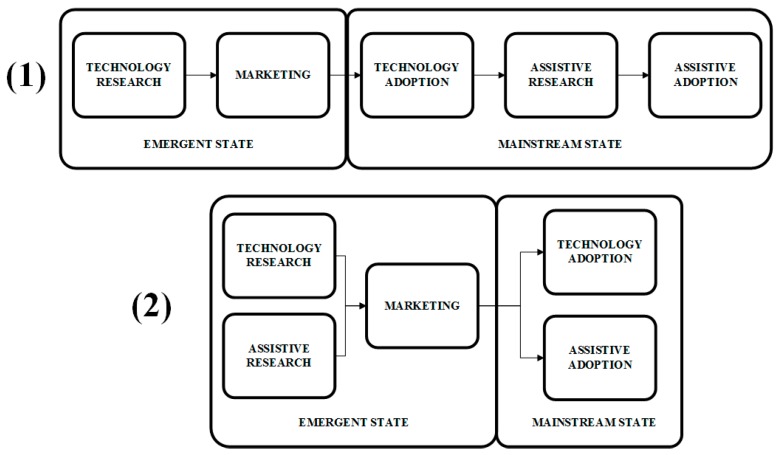
Assistive technology using widespread technologies: (1) traditional scenario and (2) proposed scenario in order to avoid differentiation with mainstream users

Lancioni stated that adaptation of commercial technology for people with cognitive disabilities whilst it is still in an emerging state (see (2) in Figure 1) can be positive in order to reduce the abandonment rate, instead of developing assistive technologies from mainstream technology once it has been already widespread adopted (see (1) in Figure 1) [72] because they will gain access to the mainstream products in a more integrated way. Commercial wearables, as the best example nowadays of emergent technology, may be a suitable vehicle for assistive technologies for cognition, in terms of inclusion and acceptance. Concretely, smartwatches are wrist-worn devices that have been widely commercialized recently, have the potential to install into society [73], but they are not fully popularized yet, so this may be the most proper time to focus the research effort of this area onto these devices. Wearing a smartwatch does not make any difference in a community where other individuals also wear smartwatches or regular watches. The difference may well be at software level, because we have mainstream hardware with the ability to implement the required assistance. 

Regarding the problem of emotional self-regulation on people with autism spectrum disorders, this paper focuses on the use of smartwatches to perform both the detection of moments of outbursts and the assistance in the form of exercises and techniques, adapted to each user, and created by means of an authoring tool implemented in the smartphone associated to the smartwatch. The hypotheses of this paper may be articulated as “Are smartwatches suitable to help people with autism spectrum disorders with their emotional regulation issues? Is this device, because of it features of pervasiveness, low intrusiveness, acceptance and usability adequate to prevent abandonment?” In order to check these hypotheses, we present and evaluate a smartwatch and smartphone system that are intended to carry out these tasks, with the advice of experts on autism spectrum disorders, and in collaboration with the Alenta center for individuals with cognitive impairment (Madrid, Spain). 

### 1.5. Taimun-Watch

#### 1.5.1. The System

As we stated in previous sections, this system is designed in order to assist people with autism spectrum disorders with their behavioral issues related to emotional regulation. This assistance is normally given by means of exercises and evidence-based practices [74], so that their caregivers and teachers provide them with materials (pictograms, instructions, verbal indications) to practice some of them, as it is usually done in case of task prompting or scheduling. However, emotional dysregulation is a pervasive issue, and hence the need of a pervasive solution. For example, if the users are walking outdoors and receive a stressful stimulus, they will not have access to the pictograms or relaxing instructions unless they remember the exercises related to them. With a pervasive assistance implemented in a smartwatch, he will have the help adapted to his needs accessible from his wrist, in a ubiquitous way. This help will be designed by their caregivers, who will be able to create, edit and send adapted exercises from their smartphone, so that both devices synchronize each time the caregiver wants to submit new changes to the smartwatch. The latter will monitor the user’s inward state to infer when the assistance should be triggered.

#### 1.5.2. Wearable Paradigm

Wearable technologies meant a shift in the interaction paradigm from the PC standards. Before, context was fixed to the very location, whereas the context related to a wearable it is always permanently changing to where the user is located in each moment [75]. Besides, we cannot forget the inclusion of the inner state of the user as context. Human-computer interaction regarding wearables (human-wearable interaction) has been already discussed in Section 1.4.2, but here we take into account the new technology paradigms that wearables entail, in terms of new devices, and the interaction between them. Concerning smartwatches, there are three types of applications:

- Standalone apps: they are executed in the smartwatch, and do not need interaction with smartphones, tablets or other supporting devices (e.g., Endomondo for Android Wear or Apple Pay for watchOS)
- Smartphone-dependent apps: they are sold as smartphone apps, but include a smartwatch module to complement the systems (as notification reader, alarms or reminders). They are usually smartphone apps that have been added smartwatch functionality in order to keep in line with the trend of new devices, and they are often called cross-device apps (e.g., Google Keep for Android Wear, iTunes for watchOS)
- Dual apps: they were designed to work together smartphone-smartwatch. None of their halves are able to work as standalone (e.g., PixtoCam for Android Wear, Slopes for WatchOS).

Normally, paradigms that imply both devices to work (smartphone-dependents or dual) are intended to be used by a single individual, who would have both devices (smartwatch in the wrist and smartphone in the pocket) so they communicate and transfer data. In addition, the programmatic structure of Android Wear apps keeps a smartphone-smartwatch application setting. Given that we want an authoring tool to create self-regulation exercises, and an assistive application for the smartwatch, we have selected the paradigm of dual applications, with the singularity of being intended for two users: the caregiver will use the smartphone part, and the individual with autism spectrum disorder will use the smartwatch, which is a suggested scheme in the literature when applying wearables to technology [76]. Nevertheless, interaction between the two devices is not needed continuously, as it happens with fitness or communication apps: they just have to synchronize data when the caregiver wants to submit new exercises and activities of self-regulation to the smartwatch. We chose Android Wear to implement the system, given the popularity, variety and feasibility of its smartwatches.

#### 1.5.3. Smartwatch: Assistive Device

As we explained, the smartwatch in this system performs two main tasks: (1) detection of temper tantrums or outbursts, and (2) display activities of self-regulation, received previously from the caregiver (smartphone). The former functionality (implicit interaction) triggers the latter (explicit interaction). In this paper, we will focus on the assistance part, hence the hypothesis. Detection has to do with the sensor capacities of the smartwatch, and implies a process of data collection, training and evaluation that may be out of the scope of this paper, besides the concept of implicit interaction, which has been discussed in earlier sections. Summarizing, we extract several features from the heart rate monitor, accelerometer and gyroscope, which helped us training a machine learning model (specifically selected to take into account the computational power of the smartwatch and the fluctuation of the sensor signals) whose classifier will run in real-time in the smartwatch. The work related to this detection part of the system is yet to be published.

In terms of explicit interaction, smartwatches have a small (circular or round) screen to display the information. It is tactile, so interaction gestures are more constrained than in smartphones [73], so the most straightforward way of interaction is through touch (short or long) and slide (vertically and horizontally), although there are works where new gestures are being studied [69]. Some of them allow audio input through microphone, and there are some recent studies about text input approaches [77,78]. However, and regarding to our users (who may find too difficult text or voice input, and self-regulation activities does not require to implement them necessarily), we will stick to the simplest, well-known ways of interaction: short touches and horizontal sliding. Although smartwatches allow to show pictures and text, their size and arrangement should be carefully selected due to the smartwatch’s screen size, and this will be a matter of content creation. 

**Figure 2:**
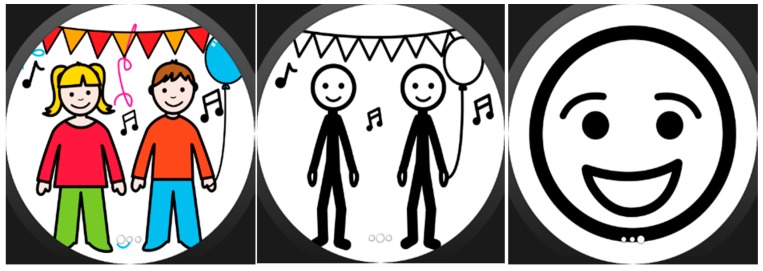
Sequence of pictograms in the smartwatch screen.

**Figure 3:**
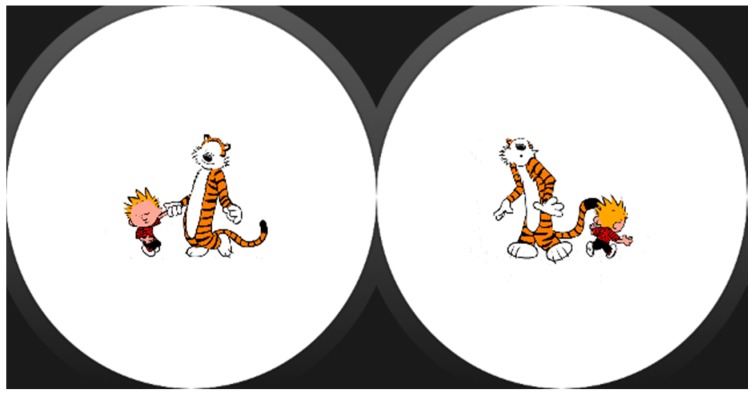
Animated GIF in the smartwatch screen.

**Figure 4:**
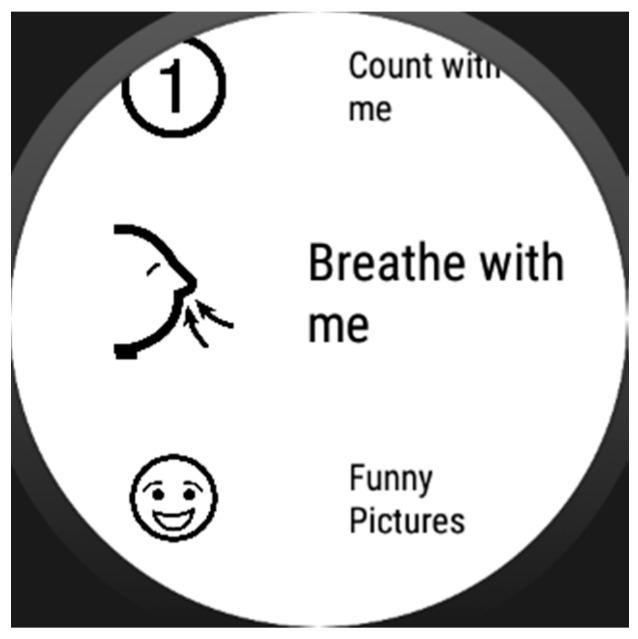
Self-regulation strategies selector.

**Figure 5:**
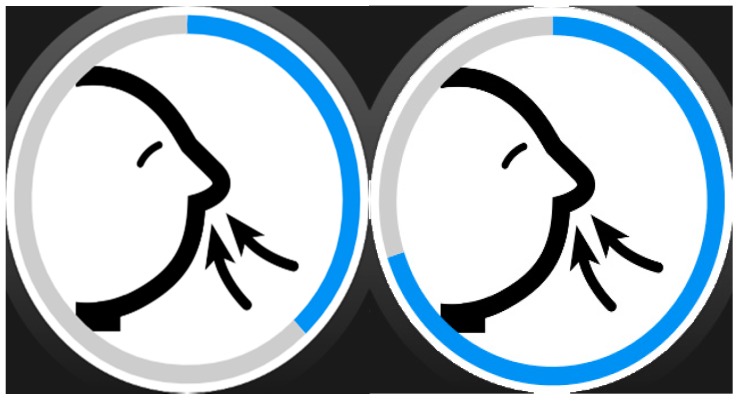
Pictograms for step-by-step breathing with round timeout.

**Figure 6:**
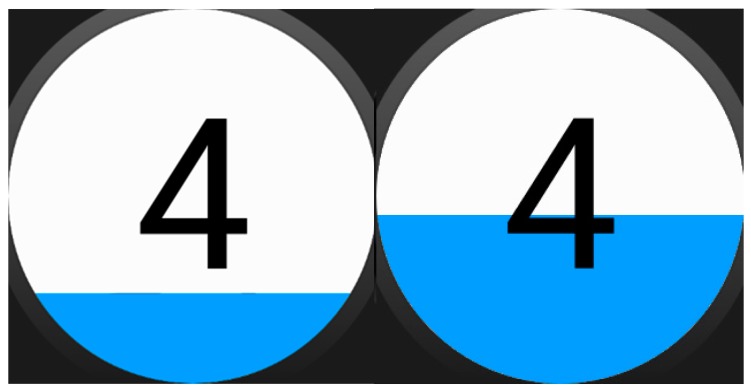
Counting strategy with screen-filling timeout.

We intended to keep the assistance format at its simplest, so assistance activities are linear sequences of “screens” that may include images (Figure 2), text, animations (GIFs, see Figure 3). Each of these sequences are called strategies composed of steps, following the nomenclature used by the experts of the center we collaborated with. However, with some users (depending on the level of cognitive impairment) may be better to let them decide which strategy to follow when a moment of need of self-regulation arise, so we decide to include selectors along with the strategies. Selectors are lists of strategies, with a preview image and a short, adapted text as description (Figure 4). Strategies can also include positive reinforcement at the end, with personalized content, and with question format: (e.g., “Did you get calm? Great!”). Depending on the configuration of the strategy (specified in the authoring tool by the caregiver), the steps may have timeouts associated, that will cause the strategy to go on with the next step (this feature would allow to implement breathing activities (Figure 5) or counting activities (Figure 6). Strategies (with steps and positive reinforcement within within) and selectors are encompassed in a global entity called regulation, which is the full piece of information that will be synchronized between smartphone and smartwatch. Regulation can be started with vibration (in many forms: short, long or intermittent) or sound depending on the configuration.

All the pictograms we used in the system have been collected from the Aragonese Portal of Alternative and Augmentative Communication [79], which is a pictogram database widely used in Spain for intervention strategies of people with cognitive impairment. 

#### 1.5.4. Smartphone: Authoring Tool

The authoring tool is implemented following two main goals: to be able to create and edit the content for the smartwatch (the assistance material) and to achieve enough level of simplicity for its use by familiars, caregivers or other people in his environment in charge of the individual. As we explained in Section 1.2.2, assistive technologies should not suppose more difficulties for the environment of the individual that traditional assistance [14], so some effort should be put in the usability of the technologic tools for them. Our choice of the Android platform is also focused on this issue: parents and caregivers of the individuals with autism spectrum disorders probably have smartphones and are used to their usage; not just for their own use, but for using assistive technologies, which have recently grown in mobile platforms [80].

**Figure 7:**
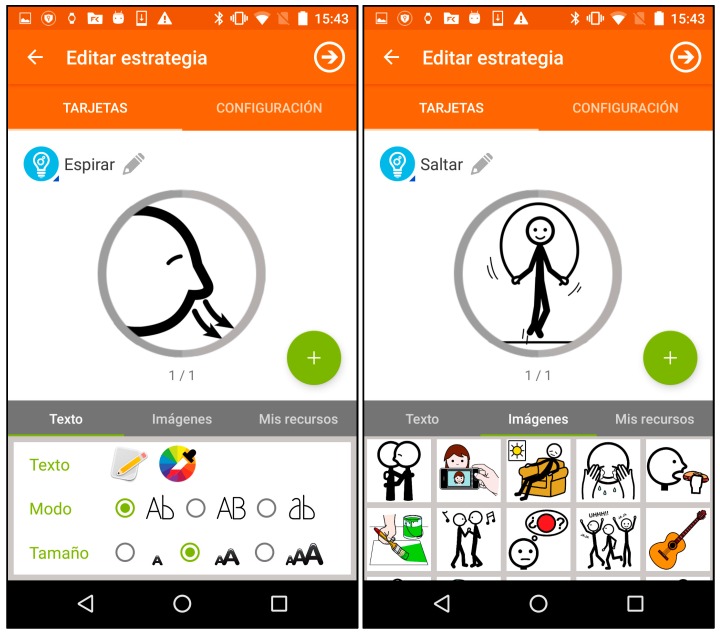
Authoring tool screenshots.

Teachers can use the in-app pictogram database to create steps and organize them into strategies, but they also can import their own resources (images and GIFs) (Figure 7). By means of a drag-and-drop interface, they can organize several strategies that will be packed into a regulation entity, and it will be sent to the smartwatch when the teacher chose to synchronize the applications. Additionally, they can configure the regulation method by selecting whether the user is allowed to select his self-regulation strategy or not (his programmed regulation is triggered automatically). This authoring tool is to be described more thoroughly in further publications, since advantages on digital editing of intervention materials goes beyond the scope of this paper.

#### 1.5.5. Self-Regulation Strategies

Emotions are the result of the cognitive evaluation of external circumstances [81], so the ways to deal with emotion-related issues, particularly when it comes to individuals with cognitive impairment, is truly diverse. This is very problematic in case of defining intervention frameworks, because emotion regulation strategies have to be adapted to each user, practically case by case. 

To design the data model behind the system, we considered the applications and advice from Dr. Quintero-Lumbreras, an expert from the Institute of Psychopediatry, who explained to us the current emotion regulation strategies used with their children. We tried to design our model so these interventions and other, multiple ones could be implemented. In Table 1 we convey how are the most used regulation strategies (crafted with pictograms in paper and direct intervention from their caregivers) implemented in the system. 

We must take into account that the majority of assistive technologies developed in order to treat behavior are based on distractors [82], so it may be difficult to distinguish between the distracting effect of the application in the smartwatch and the effects of the strategy itself.

#### 1.5.6. Use Cases

Here we will explain a use case in order to illustrate the functionality of the whole system.

“Helen is a girl with pervasive developmental disorder, not-otherwise specified, that is considered as within the autism spectrum. She’s 18 years old, and presents symptoms of dyscalculia and dyslexia. Helen is afraid of big dogs, and suffers temper outburst and explosive behaviors when a big dog is in sight, regardless of the behavior of the dog. Sometimes, she runs away to another room and does not want to go out. Even if the dog is not in sight anymore, she does not get calm, tends to cry and presents challenging behaviors to her teachers and family. They know that Helen enjoys singing songs of Spongebob Squarepants, her favorite show. She sings those songs when she feels happy, or while she is doing some activities with her friends. She likes drawing the main character, and gets very talkative when the show starts on the TV.

Adam, his caregiver, is given a smartphone and smartwatch with the app installed. He loads some images of Spongebob Squarepants and an animated GIF of the show. Then, he creates a strategy of self-regulation composed of a step showing the GIF and other steps with funny images of the show. After that, he includes some well-known pictograms for breathing that he used to practice with Helen during classes, with timeouts for each phase of breathing. He configures the strategy to vibrate at the start of the sequence. Then, he synchronizes the smartwatch with the smartphone, and it receives the configuration and the media.

Adam makes Helen wear the smartwatch, and she notices that it is very similar to the watch she used to wear, but this one have lights and is tactile. Helen’s friends do not notice anything different because they are used to see people wearing watches in the wrist. Adam configures it with a nice cover to see the hour and starts the application, which starts measuring Helen’s heart rate and movement.

Certain friend of Helen brings his new dog to class so his friends can meet him and tell them its name. Helen sees the dog, yells to Adam and runs to another classroom. Her heart rate variability is high, and she flaps her hands repeatedly − stereotypia [83]-. The application, running in the background of the smartwatch operative system, detects some patterns of outburst, and triggers the regulation strategy programmed by Adam. The smartwatch vibrates, catching the attention of Helen, and an animated GIF of Spongebob Squarepants is displayed in the screen. Helen starts singing the songs she knows, and stops thinking about the dog of her friend. Then, she sees some images of the show, and she notices that they change when she touches them. After the sequence of images, he sees the pictogram that Adam uses with her, and a round bar fills with color at the same time she breathes in and out. A message with a pictogram is shown at the end of the breathing sequence: “Well done, Helen!” She feels happy.

Helen now is more relaxed, and lets Adam go with her to the classroom, waiting for the dog to go out of the school, so she feels safe to go back with her friends. Helen was able to relax by her own and Adam did not have to participate.”

The case explained above is the optimal, targeting experience we want to achieve with the system, regarding the arguments explained in earlier sections. This is just an illustrative explanation. The real effectivity of the system is the main goal of the evaluation.

#### 1.5.7. Contribution

The idea of replacing ad hoc crafted devices with mainstream technologies has been studied previously on the literature and applied to numerous issues (for instance, smartphones performing sensing tasks to measure several parameters in test-beds [84] and physiological monitoring in real-time [85]). On the other hand, wearables (although custom ones) have been used for assistive purposes in many cases in the literature, such as visually impaired assistance with ultrasonic sensors [86] or occupational therapy [87]. A first step towards the direction of our goals would be a combination of both, that is to say, replacing custom and ad hoc devices with commercial and mainstream devices in order to achieve a goal related to assistive technologies, for the reasons discussed above. There are some studies in that direction such as the system created by Mocanu et al. [88] that aids visually impaired people to detect obstacles using smartphones or the work of Sharma et al. [64] about smartwatches that helps individuals with Parkinson monitor their activity and control their medication. 

However, our specific goal consists of employing mainstream technology to solve a particular issue within the assistive area that is the emotional self-regulation of individuals with autism. These products do not exist in the current scenario of assistive technologies, hence our contribution. 

As we have explained, the study of human computed interaction applied to wearables and the inner state of the user is in its earlier steps. Furthermore, we have not found applications that address emotional self-regulation for individuals with autism, let alone using commercial and non-stigmatizing technologies. Additionally, we combine smartphone, an already popular device, with a device in process of emergence and widespreading, whose combination had not been considered for this purpose. Thus, the main contribution of this system is its novelty and the way we applied the currently available technology to a specific issue whose proposed solutions did not involve mobile technologies nor wearables yet.

Other applications in the literature, described in the state-of-the-art related sections, propose technical advances on custom devices to address this issue. However, we argue that proposals based on devices that are not intended to reach the whole picture of users may add stigmatization to individuals with cognitive disabilities such as autism spectrum disorders. Because of that, our contribution consists of taking advantage of the existing, commercially available devices and sensors to solve a problem that has no precedent solutions using mainstream technology. Although additional sensors may enforce the inference mechanism of the system and the general effectiveness of the application, this paper argues the importance of having them included to future commercial devices instead of crafting custom ones for research purposes in single study contexts.

## 2. Materials and Methods

Although the executive dysfunction of individuals with ASD is thoroughly addressed in the psychology and pedagogic literature, its effects manifest in a broad variety of ways depending on the individual. Moreover, emotional dysregulation depends on the particular stimuli that trigger temper tantrums, outbursts and anger episodes in person with ASD.

Thus, the intervention must be highly adapted to each particular case. As we described in earlier sections, our system allows to do so. However, evaluation must also be heavily user-centered. Hence, evaluating an assistive self-regulation tool would require the evaluators to:

(a) Study the way that executive dysfunction manifests in the individual.
(b) Analyze the effects of emotional dysregulation on the individual’s behavior.
(c) Analyze the intervention that caregivers used to employ in those cases (if any).
(d) Adapt that intervention, case by case, to the tool’s format.
(e) Test the tool on the individual performing a thorough observation of the effects of the intervention.
(f) Analyze all post-session gathered data.

We have performed the steps mentioned above with two users belonging to the Alenta ASD institution. In the following subsections, we describe the experiment conducted and its results.

### 2.1. Users

The experiment has been carried out with two users from a classroom of four users with Autism Spectrum Disorders. We will refer to them as “User A” and “User B”. 

User A is 10 years old. He is an individual with ASD and mild cognitive impairment. He does not employ oral language, but alternative and augmentative communication (AAC) visual support is provided for him: pictures, pictograms and adaptable personal communicator (APC) software installed in his tablet to make petitions. User A also employs natural gestures such as taking people to places where there is something he wants. He needs people to make signs and use visual support, since his language comprehension is poor. He understands very simple, strongly contextualized instructions that involve his daily life, and visual support is always necessary for him to anticipate several situations in order to adjust his reaction and inner state about them. 

User A keeps strong, affectionate relationships with closest, well-known adults than provide him with security and things he needs. Relations with peers must be always mediated with an adult to be developed, since he does not participate spontaneously in games or group activities. Though, he tends to be curious and inquiring. Motor stereotypy with his hands, laces and cloth strips appear when he is not busy with another activity; this behavior tends to be obsessive and problematic at home. He has a low comprehension of his environment, so visual support, info panels and personal scheduling through pictograms are always needed. Additionally, due to his difficulty of behavior control, task scheduling and lack of ability, he always needs support to perform every task related to personal autonomy (pictogram sequences, adult supervision, physical support, etc.). His tolerance to frustration is rather low when he is told to keep within limits or he does not understand well what he has to do; in these moments, obsessive, disruptive behavior appear: pinching, kicking, uncontrolled laughter, pushing, and frenetic hand movement. His estimated curricular level is that of a three-year-old child.

User B is also 10 years old and an individual with ASD and mild cognitive impairment. With no oral language, he communicates through pictogram exchange, selecting pictograms from his notebook and showing it to the other person. He is able to understand simple, contextualized instructions, always with visual support in order to anticipate his reaction and inner state. User B is a smiling, affectionate individual that usually ask for the attention of the nearest adults so he can kiss them, communicate with them and, occasionally, play repetitive games. Regarding peers, he also needs adult intervention to initiate a game or to go near them. During those games, he remains silent, and does not like being forced to play or to be moved. 

User B is always in need of a well-known, structured environment where he can develop his daily activities with the aid of pictograms, info panels, and visual scheduling. He needs strong support to perform autonomous life related activities, and physical support to eat. As well as user A, his tolerance to frustration is low, and it lead him to temper tantrums when he is hungry, thirsty or feels sick. During these episodes, he tends to kick the floor, classroom chairs, cries and yells. His estimated curricular level is that of a four-years-old-child. Regarding executive dysfunction, both users present the following difficulties:

- Lack of organization skills
- Unproportioned attention to irrelevant aspects of a given task.
- Difficulty to keep an instruction in mind while inhibiting a problematic response.
- Lack of abstract and conceptual thinking.
- Literality in the comprehension of a given problem.
- Strong difficulties in the change of environment of certain tasks.
- Lack of initiative at problem solving.
- Lack of knowledge transfer between tasks.
- Inclusion of pointless activity between instructions.

Emotional self-regulation relates mainly with issues (b), (c) and (f). Paying attention to irrelevant aspects of a certain task requires intervention from the caregivers, since the individual often shows attachment to that irrelevant actions, and stopping them usually leads to outburst and anger episodes. These problematic responses also make difficult for them to retain a sequence of instructions, so stronger prompting techniques are needed. Sensitivity to environmental changes is also a main source of temper tantrums where self-regulation strategies are necessary.

These individuals are used to self-regulation strategies via pictograms. In the classroom, teachers have several pictograms ready to be used in case user A or user B (who share classroom with other two children) misbehaves due to certain stimuli. In case of user A, triggers of temper tantrums tend to be related to sensorial stimulation (moving lights, trees shadows, clouds on a sunny day, loud noises, etc.). General intervention for this user is based on distractors such as eye-catching videos on their digital board, or funny pictures with bright colors. Since those stimuli are unavoidable in an autonomous life, self-regulation support for this user must aim to get him distracted from them, using other material that also appeal to his high sensitivity. On the other hand, user B does not get altered due to overstimulation but frustration. His tantrums are scarcer than user A, but his self-regulation strategies are also narrower: apart from listening to music, it is very difficult to make the individual get calm when enraged. In the following sections, we will describe the adaptive self-regulation assistance that has been employed with each user, the devices we have used for that and the results obtained.

### 2.2. Materials

We employed LG Watch Urbane smartwatches for the experiment. They have 9-axis kinetic sensors (3-axis accelerometer, 3-axis gyroscope and 3-axis compass), PPG (heart rate monitor) and barometer. Regarding the smartphone, a Nexus 5 model was used. Both devices were linked through Android Wear interface. Each user wore a smartwatch with different self-regulation strategies loaded. 

For user A, the caregivers designed a strategy composed of a sequence of animations: a waterfall, a stream of colorful bubbles and a moving sunset. Transition between animations were configured to be by touch, and in the end of the sequence a pictogram indication of task completion was shown. For user B, caregivers chose to show a pictogram that indicated the user to listen to music. In their classroom, they always had a computer available with a pair of headphones so anyone could use them at any time. The instruction was displayed along with a round timer of 5 min. Once the time for listening to music expired, the finishing pictogram appeared. 

All pictograms were the same they used day-to-day to communicate with their caregivers, family and peers. Both users were accustomed to wear watches previously, so they did not seem particularly distracted with the fact of wearing them. Moreover, other classmates did not seem to notice any difference between them and the users, since they also worn watches of their own. This relates to the discussion about technology acceptance in Section 1.4.3, since unsatisfactory acceptance of a technologic product in a short-term experiment (such it is ours) becomes a major hindrance in its long-term usage due to the stigma associated to it. In summary, the device did not entail any rejection from the users nor their peers. The activity of user A’s application was debugged in real-time via Bluetooth, so we could monitor the technical functioning of the application during the experiment.

These two individuals shared classroom with other two ASD children who did not participate in the experiment due to bureaucratic issues and low suitability to our case (i.e., their communicative abilities were so limited that visual support or text instructions did not work for them). 

Regarding the number of users, it is important to remember that we are addressing emotional self-regulation, which is a problem strongly related to the experience and self-awareness of every individual. Hence, the effectiveness of the system has more relation to the in-depth study of its application to every individual during an extended period of time than a generic collection of data of a huge number of subjects. In other words, the in-depth dimension of the study adds more value to the study than a broad and more superficial experiment, since we are not studying the effect of an assistance product applied to an atomic phenomenon, but its effect to the continuous emotional state of the user. Conclusions are extracted within-subjects, not between subjects.

Additionally, experiments with autistic individuals should address the multi-faceted nature of autism spectrum disorders. That is to say, including a large number of individuals in a single experiment with several degrees of autism and different characteristics and skills would not imply a higher level of representativeness in your sample and would make the experiment unnecessarily complex. A scenario where a large number of individuals within the same region of the autism spectrum and similar set of abilities are studied as deep as this paper proposes is very unlikely to happen due to feasibility, cost and logistics. Nevertheless, constraining the study to a reduced set of individuals with similar set of abilities to the ones of the potential users and same location within the spectrum is feasible and favors meaningful and more concise conclusions. 

### 2.3. Methodology

The experiment was carried out during the whole class time. Given that they started at 10 a.m. and finished at 2 p.m., the system was tested 4 h a day, during nine days (in summary, 36 h of system use). Additionally, that period was often split between classroom activities and gymnastics, so we could also test the effects of the self-regulation strategies also when physical activity was involved. Two evaluators were present in all the sessions, as well as two caregivers. Evaluators took field notes, checked the debugging output and gathered all the data generated each day. Field notes contained descriptions of the individuals’ behavior and interventions from the caregivers and classmates. We also registered caregivers’ comments about the triggers that caused outbursts and behavior of the stakeholders. Regarding the stress inference, we chose to implement a simple, empiric heart rate threshold (90 bpm) based on previous monitoring of the individuals during 3 days. Although the system is prepared to perform a machine learning classification, there were two reasons to not to include it in the experiment:

- Ground truth problem: prior to the experiment, we gathered sensor data from the individuals (wearing the smartwatch), and their caregivers used a smartphone annotator software to report outbursts, temper tantrums and anger episodes. Machine learning from these data, though useful for other purposes, excludes episodes with no visible manifestation. For instance, user A showed episodes of fear and stress that manifested in subtle ways such as slightly paler skin and lip tightening: caregiver’s reports would not have noticed them during the ground truth data gathering.
- Underrepresentation: even if the ground truth data gathering was perfectly accurate, it would contain far fewer samples labeled as “stress-positive” compared to the whole set of samples of time windows where the user is calm and does not need assistance. Accuracy of any classifier built over this kind of data is hindered greatly.

Both problems are related to the inference mechanism of the application. This does not mean that there are not several approaches to stress reference in the literature. For instance, Ramos et al. [89] studied a way to take advantage of the skin conductance to infer the stress level of the individual in a real environment, but in his case ground truth is moderately easy to obtain given that standards users are capable of providing self-reports prior to the machine learning and data analysis process. Additionally, they own crafted devices that included electrodermal sensor, which is not available in the mainstream scenario. Moreover, their inference mechanism is oriented towards physical activity, which is not our case. Kim et al. [90] uses support vector machines to infer stress episodes from an input of several physiological signals successfully in real time, but it relies again on electrodermal sensors. Concerning commercial technology, Miranda et al. [91] combines Google Glasses with Zephyr wearables. However, in this case, the stigmatization of the users would be probable due to the intrusiveness of the proposed system, despite if the fact that the devices involved are available in the market. Other stress recognition studies are focused on specific contexts that are not close to ours, such us driving tasks [92].

Given that the approach of this paper is not achieving perfect accuracy but the interaction between the individual and the device and its effects on behavior (regardless of false positives or negatives), and the fact that developing a whole new inference paradigm to fit our goals would give place to further studies, we simplified the experiment as follows. 

Thresholds have been calculated empirically. Smartwatches were continuously worn by the users involved in the experiment for three whole school days prior to the actual study. Evaluators were present during these training sessions, and thresholds were tuned so that false positives would not be likely to jeopardize the experiment. It is remarkable to consider that, unlike mainstream education or mainstream students of their age, physical activity or high heart rate activity due to exercise is not much present for these students. Classroom activities are strongly based on intellectual and sensory stimulation, even physical education sessions are more focused on social interaction and self-awareness than exercising. After class, physical activity is rare, and the literature about encouraging exercise after class is abundant [93,94,95]. Thus, high values of heart rate in these kind of subjects is more likely to be caused by a stress or temper episode. Moreover, as these episodes are of emotional nature, changes of heart rate are remarkable quantitatively, so despite having tuned empirically the thresholds for both users, heart rate changes are so abrupt that even generic, lighltly tuned thresholds would work for the inference we are looking for (though these two particular thresholds were adapted to their respective subjects). 

Future versions of the system would be able to configure this threshold, as well as provide insight of suitable values for it based on the data gathered from past activity of the user. Including more complex inference mechanisms would imply to overcome the ground truth issue and the underrepresentation problem described above, as well as finding a battery-and-computation friendly method of machine learning and real-time classification for smartphones. Future devices with more computational power would be able to host an inference mechanism for this system that allows the detection to avoid false positives and to implement automatic adjustment of the detection of stress episodes and temper tantrums.

## 3. Results

Data from the smartwatches were collected and analyzed by days. Field notes were organized the same way, and we matched them to the heart rate signals. The points where heart rate signal reaches the threshold, the respective self-regulation strategy appeared on the watch. It also vibrated in order to attract the attention of the individual. 

In Appendix A and Appendix B we show the results for each day and user. As explained in earlier sections, the first half of the graphic belongs to classroom activities, whereas the second belongs to gymnastics activities. Along with the graphics, we enumerate the events that happened when the self-regulation strategies were triggered (that is to say, when the HR signal exceeded the threshold).

As we stated in earlier sections, the main points we wanted to cover during this experiment were:

1. Does the self-regulation assistance from the smartwatch help the user regain a state of calm?
2. Is the user able to interact with the device in a way that the assistance is provided and the strategies are completed?
3. What level of autonomy can they achieve with the system? Are they able to use it themselves?

### 3.1. User A

As Appendix A shows, user A’s smartwatch triggered the assistance when he misbehaved during class activities. This was to be expected, since the experts’ reports considered frustration and being forced to do something as his main sources of stress. This behavior manifests more strongly on days 3, 6 and 7. A question that arose during the experiment was if he was going to pay attention to the self-regulation instructions under these conditions. On days 3 and 6, user A not only payed attention to the smartwatch but also completed the strategy (listening to music). Evaluators agreed with the caregivers that paying attention to the smartwatch screen helped the user to evade himself from the activity he was refusing to do, or to stop fighting with certain classmate. Although his heart rate reached the threshold value several times during day 7 (thus triggering the assistance), it is noticeable how certain intervals whose values are high but do not exceed the threshold take longer to go back to lower values. However, when heart rate exceeds the threshold the two first times and the strategies are displayed on the screen, values lower more quickly, and the user regains a state of calm (the third time, as the table shows, the user ignores the watch, and his stress state remains longer). On day 6 the user also tended to discard the assistance. That may be the cause why that day’s stress peaks were repeated closely one after another (and average heart rate seems higher than other days’).

Regarding autonomy, the user needed some help to associate the pictogram prompted in the smartwatch to the act of listening to music, despite it was well-known to him. The computer with music videos was always available for him during class time, and he used it by day 4, although he need again some help on day 8. Caregivers argued that perhaps another strategy that involved more interaction with the smartwatch in place (without having to go to the computer), might have provided more insight about the effectivity of the very device in the process of self-regulation. On the second half of day 7, we can see the effects of excitement in the experiment. The user enjoyed the activity so much that his heart rate exceeded the threshold and the self-regulation assistance opened in the device. Caregivers reacted to that stating that, although enjoyment does not imply negative emotions, excessive manifestations of it may lead in some cases to tantrums or meltdowns when the stimulus that originated such excitement disappears. 

User A was able to use the system at its fullest, complete interactivity. He noticed the vibration, touched the screen, observed the pictograms and finished the sequence. Regulation strategies activated 30 times, of which 10 has been considered successful in terms of emotional self-regulation (that is to say, the heart rate lowered below the threshold and caregivers reported verbally that the user was calmed). Seven out of 20 of the rest of the system activations belong to the first three days, where they were yet learning how to use the system, and the caregivers provided guidance to complete each strategy. Seven out of the 13 remaining activations with no interaction belong to the physical impossibility to use the smartwatch because the user was doing exercise or fighting with a classmate. Finally, there were six disregarded activations due to a particularly frenzied emotional state of the user on day 7, where the user kicked, yelled, ran from the caregivers and ignored the device. Actually, the 6 activations belong to the same anger episode inasmuch as his heart rate did not lower during the tantrum. As long as the technologic assistance provided is of pervasive nature, so are the possible problems that can emerge during its use. Physical unavailability to interact, improper wearing of the watch and the presence of major distracting stimuli are expectable items in the system use scenario.

Regarding learning matters, there are two observable stages in the experiment. Days 1, 2 and 3 belong to a learning stage where the user received guidance to interact with the device and understand the strategy display method. Caregivers took the user’s hands and made him touch the screen, watch the pictograms and be aware of the timer. User A did it by himself on day 4. This is the learning plateau, that lasted until day 9. The user managed the application mostly himself, with occasional help of the caregivers to make him notice the alerts of the watch when his heart rate exceeded the threshold. The last two days, de user did not need any help to use the system, and the caregivers reported that the user was able to carry out a long-term use of it. 

As explained in earlier sections, the smartwatch repeats the programmed self-regulation strategy if the heart rate keeps above the threshold value when the instructions are finished. This only happened during the aforementioned tantrum of day 7, and on days 1 and 4, fighting with a classmate (graphically, this is represented by wider peaks of heart rate values). Hence, the user was able to self-regulate himself within the time employed by the strategy, which was 5 min. Caregivers alleged that, normally, untreated tantrums or stress episodes leaded him to more severe tantrums whose recovery would employ almost an hour of class, also requiring the attention of extra caregivers in order to redress the flow of classroom activities and affecting to his classmates’ inner state.

Thus, in a scenario where the user is familiar with the system and is physically able to interact with the smartwatch, the system did work and the user achieved himself a state of calm within a short time that allowed him and his classmates to continue regular classroom activities.

### 3.2. User B

Heart rate graphics and interaction diary can be seen in Appendix B. This individual experienced strong anxiety moments whose manifestations was not easily visible. Normally, he seemed calm. He indeed was, regarding to the graphics (expect days 7 and 8), whose average heart rate was lower and more stable than user A’s. During the nine days, user B tended to be quiet, sometimes vigilant of the behavior of other classmates, since he got easily scared of yelling or strong noises. When this happened, the heart rate raised quickly (days 1, 4, 9), although his anxiety was barely visible: he used to keep quiet and with no alarming signals but some body tightening and paleness. During these episodes, the caregivers found very difficult to get him moving, communicating and participating in classroom activities. They had not detected these episodes before, unlike the ones when the user did show anger or outburst behavior. 

On days 7 and 8, one of his classmates had an ear infection, so showed tendency to cry, yell and fight with others. User B was scared of this behavior, and manifested several signals of fear and tension: he passed almost all of the two days covering his ears. This inner state had clear effects on his heart rate: it rarely drops from the 80–90 zone. However, in the graphics we can see that the threshold (hence, the self-regulation strategies employed) acted as a limit to those heart rate peaks (that is to say, his fear episodes). Thus, the distracting component of the system appears to have helped him go through some of those episodes. From days 1 to 4, user B needed help from the caregivers to complete the strategies. The vibration and the light of the screen attracted his attention from day 1, but it was not until day 5 that he followed the self-regulation strategy himself, interacting with the smartwatch without the help of the caregivers and paying attention to the relaxing animations that they had configured for him. As we explained above, these animations were inspired on videos that helped him relax when they worked with the digital board. User B also showed tendency to cover his ears in prevention of classmates’ yelling, yet that scared him easily. At first, we wondered if having that behavior would make difficult to the user to interact with the smartwatch. Nevertheless, on days 6, 7 and 9, he lowered his arms when noticed the vibration: sometimes, he covered his ears again after that, but returned to the smartwatch after a while to see the bubbles, which was the animation he showed most engaged on. 

In summary, user B also learned to use the system successfully in terms of interaction: noticing the alert, touching the screen, observing the animations, and finishing the sequence. In comparison, he received 54 times the self-regulation alert, due to his hypersensitivity to light and sound stimuli. The majority of them occurred on days 6, 7 and 8, in which he showed particularly nervous. Twenty three out of 54 times, user B used the system successfully: he watched the animations, touched them, finished the sequence and returned to a state of calm. The remaining 29 system activations are described as follows: 15 fully-guided, five physically unable to handle, nine under major panic episodes. Given that this individual was more prone to focusing at specific task, his interaction with the device developed in a more gradual way than user A, although he needed more guided runs. As it is shown in the table, the individual started watching the device without interacting, then touched the screen shyly at further episodes, and finally managed to perform the whole strategy by himself. As with user A, during gym activities the interaction was not possible. Those nine above-mentioned system activations, as it happened with user A, occurred when the individual showed extremely scared behaviour, covering his ears, unable to focus on the smartwatch. This happened on days 7 and 8. In terms of interaction, this fact serves as an example where the behavioral problems of the user act as a barrier that jeopardize the transition between implicit interaction (i.e., the body of the individual casting physiological signals that are sensed by the device) and explicit interaction (i.e., the individual focusing on the animations and touching the screen to change from one to another) with technology (see Section 1.2). 

Regarding efficiency of the emotional regulation, we observe a clear difference between the early stages of the experiment, when the user was not familiar to the application and his heart rate peaks are wide, sustained over time, and further days, when the individual interacted with the system by himself, and his signal segments over the threshold are short. There were no chained executions of the application, as happened with user A, but his strategy did not have timers. Specifically, user B employed 30–60 s to watch the animations and get calmed down. Caregivers expected this, stating that proper intervention for this individual should be based on interaction mechanisms that distract him from the stimuli that used to perturb him. They also added that unattended minor episodes often evolved into strong panic attacks such as described before. Furthermore, we find again that in a situation of no physical conflict or extreme, rare fear episodes the system works and the user is able to recover from frequent situations of mild emotional outbursts by means of interacting with the device. This is also achieved in a short time (less than 1 min) and prevents the user from a majority of problematic outbursts.

Thus, we have found a similar, satisfying interaction results with two users whose behavior and emotional managing was rather unlike: user A shows explosive, overt temper tantrums, whereas user B’s panic attacks were hardly noticeable unless the caregiver payed special attention at his behavior. These cases cover a wide range of individuals with ASD in terms of how alexithymia and emotional dysregulation manifest behaviorally. In both cases, there was a majority of occasions where the system was able to help the individual control their stress episodes, caused by a variety of stimuli, excluding the learning phase of the experiment. In the remaining cases, the assistance from the smartwatch was either not applicable or an unusual episode of major temper tantrum happened.

## 4. Conclusions

Emotional dysregulation entails a meaningful impact of the long-term quality of life of people with ASD. Anxiety, depression, explosive behaviors and inadaptability are common effects on these individuals if untreated. Traditional support for these matters is based on printed pictures, activities within class and direct intervention of the caregivers using the mentioned resources. However, these methods are unfeasible, time-consuming, hardly customizable and not pervasive. 

Inability to manage and recognize their own emotions is what leads to these difficulties. It is called alexithymia, and recent technology products in the literature are meant to address this characteristic of individuals with ASD, inasmuch as their inner state can be read in alternative ways (physiological signals, facial expressions) and proper intervention might be provided. 

Nonetheless, including technology in the daily life of a person with ASD, as for individuals with cognitive impairment, is a sensitive issue. Long-term acceptance of assistive devices is unlikely if they result in stigmatization, cumbersome management or low customization possibilities.

In this paper, a system for the self-regulation of individuals with ASD is presented and evaluated. It is composed of a smartwatch to detect the user’s inner state and display the self-regulation strategies and a smartphone to create and customize such interventions. The wearable device monitor the user’s heart rate and displays the intervention when that signal exceeds a configurable threshold. Smartwatches do not stigmatize the wearer, since they resemble well-known devise (regular watches), its content is fully customizable by means of the authoring tool and their functioning is based on the Android paradigm, to whom many technology owners are used. 

The system is also feasible (paper resources and printed pictures are no longer needed), time-saving (its content creation is straightforward and visually attractive), customizable and pervasive (the user receives the intervention anywhere and anytime if wearing the smartwatch). We studied its effect during nine days of use of two individuals with significant behavioral problems. Their classmates did not notice difference in them for the fact of wearing smartwatches (i.e., no stigmatization), and the system helped them control the majority of their stress episodes within minutes. 

Being able to manage a big number of minor tantrums is highly positive for these individuals, yet long-term issues are based on the accumulation of episodes of exposure to unpleasant, self-harming sensations that are not channeled properly due to their alexythimia. This is why our system ensure a major improvement on the quality of life of people with ASD and emotional dysregulation problems, since small-scale intervention applied to the daily life of these individuals prevents long-term behavioral issues to develop.

## 5. Future Work

Some major improvements can be implemented in the system so it is fully generalizable and scalable. The main technical upgrade should be the inference mechanism of stress episodes. As we exposed previously, it is arguable that machine learning system based on ground truth from the caregivers’ observation excludes temper tantrums with no clearly visible manifestation. Nevertheless, it is interesting to study new ways of ground truth extraction such as mixing external reports from the caregivers and supervised sensor data or applying advanced preprocessing techniques to lessen the problem of imbalanced data. Automatic stress recognition algorithms can be also adapted to ASD characteristics in order to be applied to the system. Most of these algorithms rely on data from electrodermal sensors, so future smartwatch models that include it may entail a huge advancement on stress inference from physiological signals.

Related to this, with the data retrieved from the smartwatch after each synchronization, caregivers should be able to see the effects of the strategies they had implemented. This may be possible if this information is presented in a legible way, so a future improvement of the smartphone part of the system could be to integrate a data visualization software. 

Finally, further experiments with more users would provide more generalizable data and raise other problematic issues that may have not appeared in this experiment. Additionally, testing the smartphone authoring tool through questionnaires for the caregivers would complement the evaluation of the system with usability information.

## Figures and Tables

**Table 1:** Traditional intervention strategies and their implementation in the system.

Self-Regulation Strategy	Implementation
Counting numbers	Sequence of (numbers/picture representation of numbers/picture representation of quantities). It has timeout if the counting is automatic, it does not if the user is intended to touch the screen with each number
Sitting and relaxing	Sequence of pictograms telling the user to sit and relax
Grasping a certain object	Sequence of pictograms telling the user to look for the object and grasp it
Going for a walk	Sequence of pictograms and animated GIFs telling the user to walk or run
Asking an adult for help	Sequence of pictograms telling the user to look for an adult, combined with other timed strategy meanwhile
Two-phase breathing	Sequence of pairs of timed pictograms with indications of breathing in and out
Asking for a hug	Sequence of pictograms telling the user to look for an adult and asking him for a hug, combined with other timed strategy meanwhile
Looking funny/relaxing pictures	Sequence of such images, with or without timing

## Appendix A

	1. Misbehaves during classroom activities. Frustration. 2. Temper tantrum, gets hit by a classmate.
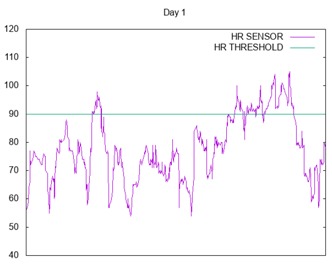
	1. Fights and chases classmate. 2. Wants to go to the bathroom. Gets frustrated because of not being able to communicate.
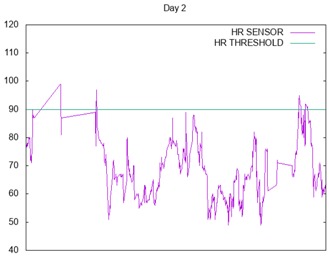
	1. Misbehaving. Stares at the watch carefully 2. Misbehaving. Touches the watch screen. 3. Misbehaving. Touches the screen and listens to music. Touches the screen again and finishes the strategy. 4. Same as (3), during gymnastics
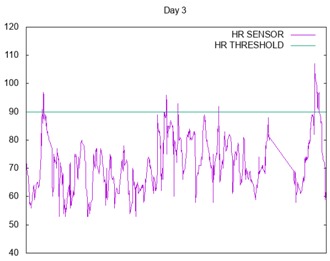
	1. Fights classmate. Does not look at the watch. 2. Chases classmate. Looks at the watch. Listens to music and finishes the strategy. 3. Gets frightened of classmate’s outburst. Looks at the watch. Discards the strategy by touching quickly the screen.
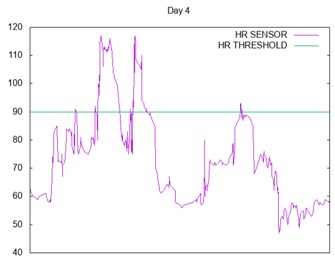
	1. No temper tantrums. Seems happy and works well. Seeks caregivers attention repeatedly.
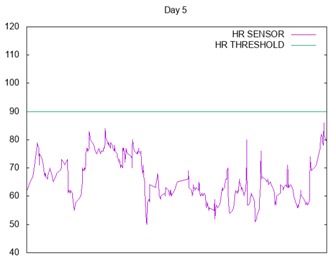
	1. Continuous misbehaving. Looks at the watch, listens to music and finishes the strategy. 2. Misbehaving during gymnastics. Discards the strategy by touching the screen quickly. 3. Refuses to do some physical activities. Looks at the watch for a while, then discards the strategy.
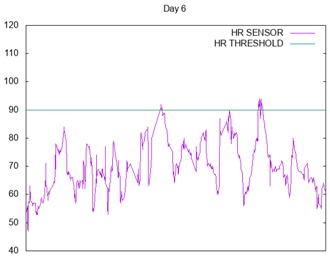
	1. A classmate chases him and tries to touch his watch. Cries and looks briefly at the watch. 2. Refuses to do a classroom activity. Looks at the watch and is put to listen to music. 3. Seems continuously excited because they are doing a physical activity concerning animals, which he seems to enjoy. Ignores the watch.
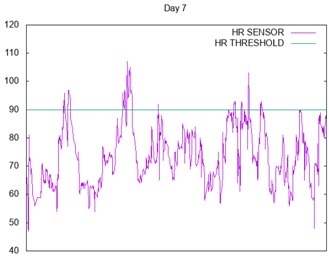
	1. Anger episode when the caregiver makes him wear the watch. Looks at the strategy, is put to listen to music and finishes the strategy. 2. Wants a candy, and the caregiver gives it to him. Ignores the watch.
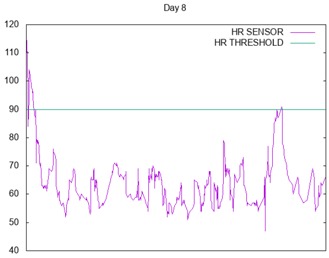
	1. Not noticeable trigger. Touches the screen and discards the strategy.
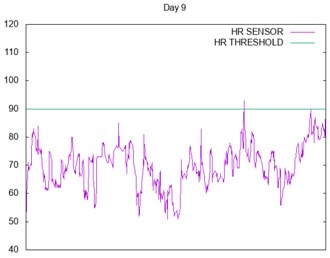

## Appendix B

	1. Gets frightened of classmate’s yelling. Follows the watch strategy with the help of the caregiver. 2. Temper tantrum: patting, kicking, covers his ears. Follows the strategy with the caregiver. Looks at it quietly. 3. Gets frightened of turning off the gyms lights. Follows the strategy with the caregiver.
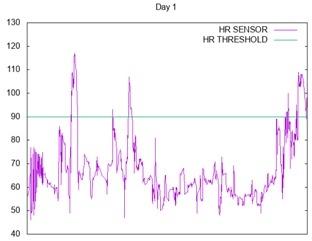
	1. Dislikes wearing the watch. Manages to loosen it. 2. Caregiver makes him wear it again. Mild complain. Watches the screen. 3. Gets frightened of classmate’s yelling. Watches and touches once the screen. 4. Watch gets loosened during physical activity. Complains and watches the screen. Notices the bubbles.
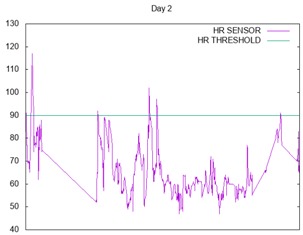
	1. Complains of watch wearing. Follows the strategy with the caregiver. 2. Gets frightened of classmate’s outburst and yelling. Looks at the screen, touches it, notices the bubbles.
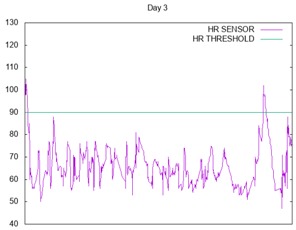
	1. Trees’ shadows scare him. Follows the strategy with the caregiver. Looks for a moment at the bubbles and the sunset. 2. Caregiver makes him climb the gym wall bars. Gets frightened of the height. Cannot use the watch. Seems scared for a long interval of time.
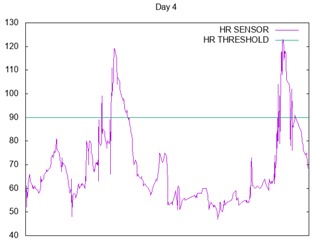
	1. Classmates move tables and chairs; the noise scares him. Looks and touches the watch’s screen. Looks at the sunset animation for a little while. 2. Classmate’s outburst. Ignores the watch. Transition between classroom and gym seems to disturb him.
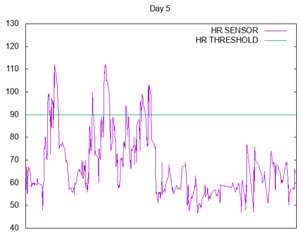
	1. Classmate yells repeatedly. Covers his ears, looks at the screen when the watch vibrates, touches it. Looks for a while at the bubbles. Finishes the strategy. 2. Same as (1), but switches between covering his ears and looking at the bubbles animation.
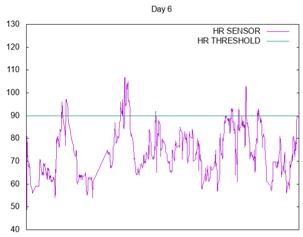
	1. Classmate yells very often, and people enter the classroom repeatedly. Strategies are triggered repeatedly. Most of the times, he looks and touches the screen. He seldom ignores it, but seems agitated anyway.
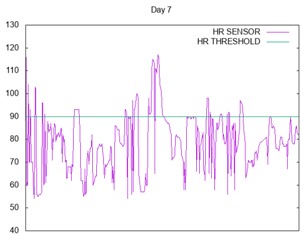
	1. Same as (Day 7), but ignores the watch more often. During gymnastics class, he seems more relaxed.
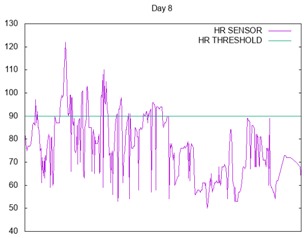
	1. Classmate yells, and gets frightened of it. Follows the strategy. Keeps the bubbles on the screen for a while. Finishes the strategy. 2. On two occasions, seeks the caregiver’s attention and points to the watch. Seems to be engaged with the bubbles.
